# Deepak Malviya: President’s message

**DOI:** 10.4103/0019-5049.76559

**Published:** 2011

**Authors:** Deepak Malviya

**Affiliations:** President. ISA 2011 Department of Anaesthesiology and Critical Care Medicine, B.R.D. Medical College, Gorakhpur, UP 273013, India. E-mail: drdm58@gmail.com


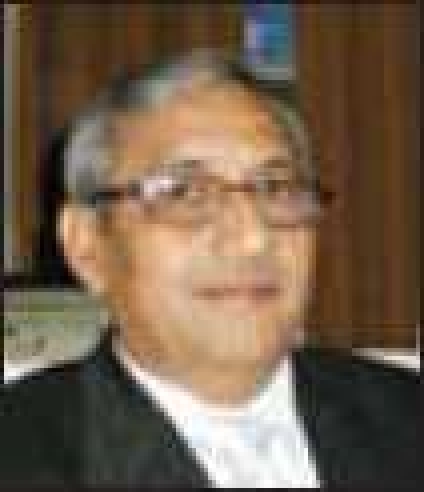


Dear Members,

With great pleasure, I greet you all in the beginning of New Year 2011. I wish you and your family health, happiness and prosperity. My sincere gratitude to all of you for giving me the opportunity to serve as President of Indian Society of Anaesthesiologists (ISA). I am overjoyed to express my great sense of gratitude for unanimously electing me to the highest chair. I will try to fulfil all the responsibilities which you are entrusting on me.

The ISA always believes in serving the society that we live in. In future, the association will be much bigger than it is now. More importantly, the association will be a trendsetter in education and training, and will be strong enough to protect the interests of all our members.

Our vision for the society is to play a very active role in making high-quality health-care system accessible and affordable to all in our country.

The task ahead of us will be

1. to assist our members to improve the knowledge and skill through ISA sponsored workshops and CMEs;
2. to ensure full functioning of the academic wing of ISA, i.e. Indian College of Anaesthesiology;
3. to support our fellow members in their personal moment of need;
4. to establish various protocols in anaesthesiology and emergency medicine for the Indian scenario; and
5. WHO’s “*safe surgery saves lives*” requires safe anaesthesia practice and ISA will collaborate with WHO. ISA is also collaborating with WHO in Global Pulse Oximetry Project.

The year 2011 will be full of challenges and commitments. This year, we are hosting SAARC Congress at Bangalore which is a matter of honour and prestige for ISA and our Nation. We accept the challenge and will leave no stone unturned to make this mega academic event successful.

Friends, we have just begun. The opportunities in front of us are limitless and there is a lot that needs to be done. Whatever we commit, we will do it. Our success depends on how focussed we are. Our willingness, commitment and personal responsibilities are the key stones for our success. As we move into the new year, I have no doubt that with well-concerted and integral efforts, we will succeed.

Once again, I wish you and your loved ones a Safe, Healthy and Happy New Year 2011!

Jai Hind!
Long Live ISA!
Warm regards,

